# Neuroimaging assessment of pediatric cerebral changes associated with SARS-CoV-2 infection during pregnancy

**DOI:** 10.3389/fped.2023.1194114

**Published:** 2023-05-24

**Authors:** David Alves de Araujo Junior, Felipe Motta, Geraldo Magela Fernandes, Maria Eduarda Canellas De Castro, Lizandra Moura Paravidine Sasaki, Licia Pacheco Luna, Thalys Sampaio Rodrigues, Patricia Shu Kurizky, Alexandre Anderson De Sousa Munhoz Soares, Otavio de Toledo Nobrega, Laila Salmen Espindola, Alberto Moreno Zaconeta, Ciro Martins Gomes, Olindo Assis Martins-Filho, Cleandro Pires de Albuquerque, Licia Maria Henrique da Mota

**Affiliations:** ^1^ Department of Medicine, University of Brasilia (UnB), Brasilia, Brazil; ^2^ Hospital Universitario de Brasília (HUB), Brasilia, Brazil; ^3^ Medical Sciences, University of Brasilia, Brasilia, Brazil; ^4^ Department of Medicine, Austin Health, University of Melbourne, Melbourne, VIC, Australia; ^5^ Russell H. Morgan Department of Radiology and Radiological Science, Johns Hopkins University School of Medicine, Baltimore, MD, United States; ^6^ Instituto René Rachou, Fundação Oswaldo Cruz (FIOCRUZ-Minas), Belo Horizonte, Brazil

**Keywords:** COVID, SARS-CoV-2, pregnancy, neonatology, ultrasound, elastography, neuroimaging

## Abstract

**Background:**

SARS-CoV-2 infection and perinatal neurologic outcomes are still not fully understood. However, there is recent evidence of white matter disease and impaired neurodevelopment in newborns following maternal SARS-CoV-2 infection. These appear to occur as a consequence of both direct viral effects and a systemic inflammatory response, with glial cell/myelin involvement and regional hypoxia/microvascular dysfunction. We sought to characterize the consequences of maternal and fetal inflammatory states in the central nervous system of newborns following maternal SARS-CoV-2 infection.

**Methods:**

We conducted a longitudinal prospective cohort study from June 2020 to December 2021, with follow-up of newborns born to mothers exposed or not exposed to SARS-CoV-2 infection during pregnancy. Brain analysis included data from cranial ultrasound scans (CUS) with grayscale, Doppler studies (color and spectral), and ultrasound-based brain elastography (shear-wave mode) in specific regions of interest (ROIs): deep white matter, superficial white matter, corpus callosum, basal ganglia, and cortical gray matter. Brain elastography was used to estimate brain parenchymal stiffness, which is an indirect quantifier of cerebral myelin tissue content.

**Results:**

A total of 219 single-pregnancy children were enrolled, including 201 born to mothers exposed to SARS-CoV-2 infection and 18 from unexposed controls. A neuroimaging evaluation was performed at 6 months of adjusted chronological age and revealed 18 grayscale and 21 Doppler abnormalities. Predominant findings were hyperechogenicity of deep brain white matter and basal ganglia (caudate nuclei/thalamus) and a reduction in the resistance and pulsatility indices of intracranial arterial flow. The anterior brain circulation (middle cerebral and pericallosal arteries) displayed a wider range of flow variation than the posterior circulation (basilar artery). Shear-wave US elastography analysis showed a reduction in stiffness values in the SARS-CoV-2 exposed group in all analyzed regions of interest, especially in the deep white matter elasticity coefficients (3.98 ± 0.62) compared to the control group (7.76 ± 0.77); *p*-value < 0.001.

**Conclusion:**

This study further characterizes pediatric structural encephalic changes associated with SARS-CoV-2 infection during pregnancy. The maternal infection has been shown to be related to cerebral deep white matter predominant involvement, with regional hyperechogenicity and reduction of elasticity coefficients, suggesting zonal impairment of myelin content. Morphologic findings may be subtle, and functional studies such as Doppler and elastography may be valuable tools to more accurately identify infants at risk of neurologic damage.

## 1. Introduction

Maternal infection with SARS-CoV-2 during pregnancy may expose the fetus to both direct and indirect systemic effects triggered by the virus (1). The consequences of the maternal and fetal inflammatory response with the production of potentially cytotoxic cytokines, in addition to the effect of the use of antiviral medications, have not been adequately studied to date (1, 2).

There is evidence that vascular complications may result from the potential hyperactivation of inflammatory factors and coagulation system dysfunction, particularly D-dimer and platelet abnormalities, increasing the risk of cerebrovascular disease, myelination defects, and hypoxic-ischemic encephalopathy following exposure to SARS-CoV-2 (3, 4). The potential consequences of changes in intracranial blood flow dynamics and cerebral hypoxia, mediated by systemic inflammatory response syndrome (SIRS), are still poorly elucidated in the pediatric age group (5). This study aimed to investigate the effects of maternal SARS-CoV-2 infection on the brains of infants exposed to SARS-CoV-2 infection during pregnancy, focusing on brain morphological changes, intracranial blood flow dynamics, and parenchymal composition/stiffness analysis. Additionally, we sought to assess the clinical and neurodevelopmental outcomes of newborns following maternal SARS-CoV-2 infection.

## 2. Methods

### 2.1. Study design and population

A prospective, comparative, and analytical cohort study was conducted with the follow-up of newborns born to mothers exposed or not exposed to SARS-CoV-2 infection during pregnancy. The study population consisted of 219 children, of whom 201 were in the group of newborns born to women infected by SARS-CoV-2 at different stages of pregnancy. The control group consisted of 18 newborns born to women who remained serologically negative for SARS-CoV-2 until the end of the neonatal period. Study recruitment was from May 2020 to June 2022, during the COVID-19 pandemic, with planned clinical, neurological, and psychomotor follow-up until December 2022. Clinical follow-up was performed monthly until 6 months of age and then quarterly until 24 months of age. A global pediatric assessment and a neuro-psychomotor development diagnostic scale (Bayley III scale) were administered quarterly. Neuro-ultrasonography, color/spectral Doppler, and shear-wave elastography studies were performed at 6 months of adjusted chronological age, and follow-up evaluation was completed 4 weeks later for the abnormal cases. Detailed maternal clinical characteristics were also prospectively collected.

The case group included exposed newborns born to mothers infected by SARS-CoV-2 during pregnancy (RT-PCR or positive IgM). The control group included unexposed neonates with no maternal infection with the SARS-CoV-2 virus during pregnancy, no symptoms, and negative IgG and IgM serology at the end of pregnancy. Unexposed control subjects had negative IgG serology at 6 months of adjusted chronological age. Exclusion criteria for the study sample were evidence or confirmation of genetic syndromes; suspected or confirmed other congenital infections, such as toxoplasmosis, syphilis, rubella, herpes, Chagas, and Zika; discontinuation of clinical follow-up before the age of 2 years.

### 2.2. Neuroimaging data

The study groups were evaluated for morphometric, hemodynamic, and cerebral tissue elasticity parameters using high-frequency ultrasonography. A cranial ultrasound scan (CUS) was performed through the anterior fontanelle at 6 months of adjusted chronological age and repeated 4 weeks later at a follow-up exam in case of abnormal findings at the first CUS. Further examinations were planned thereafter, if indicated, according to an individualized schedule based on the persistence of abnormal findings and their clinical correlation. Only data from the first ultrasound scan of each infant were considered for the purpose of statistical analysis. All CUS were performed or supervised by the same operator (DA). A Philips Affiniti 70 ultrasound system, equipped with a 5–7.5 MHz convex probe and a 5–18.0 MHz linear probe, was used for CUS studies, divided into the following modalities:

- Cranial ultrasound scan and Doppler of the intracranial arteries—pericallosal artery, middle cerebral artery, and basilar artery; Doppler velocimetry data were analyzed for each individual artery. Absolute values of resistance (RI) and pulsatility index (PI) for blood flow were compared in both groups (exposed vs. control). Images were obtained at the same standard windows over the anterior fontanelle, temporal bone, and suboccipital zone.
- Elastography of the brain parenchyma: scans were performed using ARFI (Acoustic Radiation Force Impulse) and SWE (Shear-wave Elastography) software, which is directly integrated into the ultrasound system where the shear wave is located, allowing the operator to select the region of interest (ROI) for measurement in B-mode and in real-time. The ROIs were divided as follows: deep white matter (DWM), superficial white matter (SWM), basal ganglia (BG), represented by caudate nuclei and thalamus, corpus callosum (CC), and cortical gray matter (CGM) in the frontal lobe. All measurements were repeated at three different locations in the same type of zone and the same slice of view. The archived data represent the mean value of the measurements.

Tissue elasticity was estimated, and the velocity of the shear wave in the brain parenchyma was calculated from the displacement of transverse waves, where the velocity of the shear wave is directly proportional to the local tissue stiffness. The results were expressed in meters per second (m/s) and automatically converted to kilopascals (KpA), with the shear wave propagation velocity being proportional to the square root of the tissue elasticity [*E* = 3pc^2^].

### 2.3. Data storage

Study records were stored using alphanumeric codes in the REDCap (Research Electronic Data Capture) platform, with access restricted to approved research personnel.

### 2.4. Statistical analysis

Continuous variables were described as mean and standard deviation or median and interquartile range (IQR), as appropriate. Categorical variables were expressed as frequencies and percentages. The prevalence of total abnormalities was evaluated for each category and compared with its prevalence in the control and SARS-CoV-2-exposed groups. A Mann-Whitney *t*-test or ANOVA were used to compare the mean values of imaging parameters.

The neuroimaging parameter means were compared between groups (SARS-CoV-2 exposed vs. controls) using an analysis of covariance (ANCOVA) model. In the ANCOVA model, the neuroimaging parameter measures (RI and PI for Doppler; “E” coefficient/Young's modulus for elastography ROIs) were considered dependent variables, the group (SARS-CoV-2 exposed vs. unexposed) was considered the independent variable, and the measures of GA (gestational age) and BW (birth weight) were considered covariates. A significance level of *p* < 0.05 was considered. Study participants were also grouped into two different categories based on their GA (pre-term and term, considering the cut-off at 37 weeks) and BW (low birth weight and adequate birth weight, considering the cut-off at 2,500 g).

A Cochran-Armitage trend test and Pearson correlation were used to evaluate trends or associations of results between Doppler and cranial ultrasound scans. *p*-value < 0.05 was considered significant. Analyses were performed using SAS v. 9.4 (SAS Institute, Inc., 2016).

### 2.5. Ethical approval and informed consent

The study was approved by the Research Ethics Committee of the School of Medicine of the University of Brasilia (Certificate Number C.A.A.E 32359620.0.0000.5558). The protocol was also registered in the Brazilian Registry of Clinical Trials. All pregnant women participating in the study gave informed consent. Likewise, the participation of the children in the pediatric arm required the signed, informed consent of their mothers. The 6-month reports on the status of the study and its partial results are available to the Institutional Research Ethics Committee and can be consulted upon request.

## 3. Results

### 3.1. Overview of enrolled subjects

The initial screening included 295 volunteers, sorted by hospital unit, gender, age, and trimester of maternal infection. Two subjects withdrew from the study after the initial phase. Of the 293 subjects evaluated, 74 were excluded due to: (a) loss of follow-up (*n* = 72) and (b) diagnosis of congenital infection (*n* = 2). After exclusions, the total sample included 219 participants, consisting of 201 subjects with documented maternal SARS-CoV-2 infection [exposed group; birth age = 39 ± 2.9 weeks (mean ± SD); 56.6% female] and 18 subjects not exposed to SARS-CoV-2 infection [control group; birth age = 39 ± 2.8 weeks (mean ± SD); 53.0% female]. Gestational age at birth ranged from 33 to 42 weeks (mean 38.1 ± 1.8 weeks), and birth weight ranged from 1,525 to 4,418 g (mean 3,127 ± 535 g).

Concerning maternal diseases prior to pregnancy being affected by COVID-19, 16 patients (7%) had a previous history of systemic arterial hypertension, 3 (1.3%) reported pre-eclampsia, and 15 (6.8%) were affected by pregestational diabetes. A total of 15 patients had a history of pulmonary disease (6.8%), including asthma, and six had heart disease (2.7%).

Considering the clinical characteristics of the control group, the mean age at birth is 38.8 weeks of gestation, with a standard error of ±0.41 w; mean birth weight for controls is 3,277 g, with a standard error of ±107 g; mean head circumference is 35 ± 0.1 cm. The median and interquartile range (IQR) values for the first- and fifth-minute APGAR scores in the control group are 8 (IQR: 7–8) and 9 (IQR: 9–9), respectively. With our cut-offs of 37 weeks for prematurity and 2,500 g for low birth weight, we have 16.6% (3) pre-term and 11.1% (2) low-birth weight individuals in the control group; the case group has similar frequencies with 14.8% (26) pre-term and 16.5% (29) low-birth weight individuals. Among the comorbidities found within the groups, the most frequent were anemia, bronchospasm, malnutrition, obesity, rhinitis, dermatitis, cow's milk protein allergy (CMPA), and gastroesophageal reflux disease (GERD). A supplemental table in Appendix C is provided for reference, demonstrating that groups display a similar profile of comorbidities.

### 3.2. Findings by imaging modality

#### 3.2.1. Grayscale ultrasonography (structural US)

An association was found between maternal SARS-CoV-2 infection and white matter involvement in their children, with increased echogenicity in grayscale studies. Among the 201 examinations performed in the case group, 18 examinations showed abnormalities in B-mode analysis (8.9%), with deep white matter disease in the totality of these 18 abnormal cases (100%). To a lesser extent, we also saw mild alterations in the basal ganglia (caudate nuclei and thalamus), with abnormal caudothalamic echogenicity in 2 (11.1%) of 18 abnormal B-mode cases, concurrent with the deep white matter findings.

Supplementary Figure A1 summarizes the three main planes for cranial image acquisition and ultrasonographic analysis of deep white matter changes. It also shows CUS B-mode and Doppler velocimetry studies, analyzing three major intracranial arteries (the middle cerebral, pericallosal, and basilar arteries).

An equally significant finding of the morphometric US studies was the persistence of increased echogenicity in the affected areas at the routine second-look ultrasound study, performed 4 weeks after the initial study, in all the abnormal cases. On re-evaluation, it was possible to characterize the clear extension of the affected areas, with additional abnormalities in the basal ganglia—in total, the caudate nuclei and thalami. It is also noteworthy that there were no individuals in the control group (18 out of 219) with grayscale ultrasound alterations.

#### 3.2.2. Hemodynamic abnormalities (Doppler velocimetry)

In the exposed group, 21 out of 201 (10.4%) subjects presented with abnormal hemodynamic patterns, showing a reduction in the resistance (RI) and pulsatility (PI) indices in the blood flow of the major intracranial arteries. We conducted separate analyses of three main intracranial arteries: the middle cerebral artery, the pericallosal artery, and the basilar artery, the former two representing hemodynamic parameters for the anterior intracranial circulation, and the basilar artery velocimetry as an estimate of posterior circulation flow data.

Supplementary Figures A2–A4 demonstrate the Doppler velocimetric scan with spectral curves for the analysis of the flow of three major intracranial arteries (middle cerebral, pericallosal, and basilar arteries).

A significant trend of reduction in both resistance and pulsatility indices of arterial intracranial flow in SARS-CoV-2-exposed children were observed for both anterior and posterior circulation arteries, which was positively correlated with the severity of maternal infection. Significant decreases in RI and PI were found in cases of critical SARS-CoV-2 gestational infection, with mean PI values of 1.09 for the MCA (middle cerebral artery), 0.98 for the PA (pericallosal artery), and 1.04 for the BA (basilar artery).

Table 1 shows the neuroimaging parameters according to the severity of maternal infection (COVID-19 categories according to WHO classification).

**Table 1:** Imaging parameters distributed according to the severity of maternal infection (COVID-19 categories according to WHO classification).

Variable^a^	COVID-19 severity scale—WHO			ANOVA	Multiple comparisons *p*-value*		
	Mild (*n* = 165)	Severe (*n* = 23)	Critical (*n* = 8)	*p*-value	Mild to severe	Mild to critical	Severe to critical
MCA RI	0.77 ± 0.09	0.75 ± 0.10	0.64 ± 0.14	0.0011	0.8394	0.0010	0.0260
MCA PI	1.64 ± 0.50	1.47 ± 0.38	1.09 ± 0.45	0.0039	0.3789	0.0064	0.1713
Pericallosal RI	0.70 ± 0.08	0.68 ± 0.10	0.61 ± 0.09	0.0062	0.7004	0.0070	0.1185
Pericallosal PI	1.29 ± 0.31	1.19 ± 0.25	0.98 ± 0.24	0.0113	0.4744	0.0181	0.2833
Basilar RI	0.72 ± 0.07	0.69 ± 0.09	0.64 ± 0.09	0.0032	0.1692	0.0093	0.3330
Basilar PI	1.34 ± 0.29	1.24 ± 0.28	1.04 ± 0.28	0.0121	0.4389	0.0208	0.3229

MCA, middle cerebral artery; RI, resistance index; PI, pulsatility index.

^a^values expressed as mean ± standard error.

*p-values for multiple comparisons adjusted with Bonferroni correction.

When both analyses, mode-B ultrasound, and Doppler scan findings, were integrated and cross-matched with the categories of maternal infection severity, a positive correlation of abnormal neuroimaging results that increased proportionally with the severity of maternal infection, and a peak of abnormal neuroimaging results in children whose mothers had critical SARS-CoV-2 infection during pregnancy could be identified. These data are summarized in Table 2.

**Table 2:** Neuroimaging parameters are distributed according to the COVID-19 severity scale—WHO classification.

Variable^a^	COVID-19 severity scale—WHO^a^			Pearson correlation (CI 95%)	*p*-value*
	Mild (*n* = 166)	Severe (*n* = 23)	Critical (*n* = 8)		
Intracranial Doppler				0.23 (0.06; 0.40)	<0.001
Abnormal	11 (6.63)	5 (21.74)	5 (62.50)		
Normal	155 (93.37)	18 (78.26)	3 (37.50)		
Ultrasonography				0.24 (0.06; 0.42)	<0.001
Abnormal	9 (5.42)	5 (21.74)	4 (50.00)		
Normal	157 (94.58)	18 (78.26)	4 (50.00)		

^a^ Values expressed in frequency (%).

^*^ *p*-value calculated with the Cochran-Armitage trend test.

A second trend in the hemodynamic data was identified in this analysis, related to the duration of SARS-CoV-2 infection during pregnancy. A significant reduction in both the resistance and pulsatility indices of intracranial arterial flow positively correlated with the last trimester of maternal SARS-CoV-2 infection, as shown in Table 3.

**Table 3:** Neuroimaging parameters (ultrasound B-mode and Doppler analysis) distributed according to the trimester of SARS-CoV-2 infection during pregnancy.

Variable^a^	Gestational trimester of SARS-CoV-2 infection^a^					
	1st (*n* = 27)	2nd (*n* = 58)	3rd (*n* = 95)	Peripartum (*n* = 21)	Pearson Correlation (CI 95%)	*p*-value*
Intracranial arteries Doppler					0.43 (0.32; 0.54)	<0.001
Abnormal	0 (0.00)	1 (1.72)	6 (6.32)	14 (66.67)		
Normal	27 (100.00)	57 (98.28)	89 (93.68)	7 (33.33)		
Ultrasound B-mode					0.41 (0.30; 0.53)	<0.001
Abnormal	0 (0.00)	1 (1.72)	4 (4.21)	13 (61.90)		
Normal	27 (100.00)	57 (98.28)	91 (95.79)	8 (38.10)		

^a^ Values expressed in frequency (%).

^*^ *p*-value calculated with the Cochran-Armitage trend test.

The hemodynamic evaluation data show a significant correlation between the resistance/pulsatility indices in the main intracranial arteries and the trimester of maternal infection, with the highest proportional frequency of abnormal results observed in cases of peripartum infection (defined as a period equal to or less than 14 days between infection with SARS-CoV-2 and the date of delivery). Among the pregnant women infected during this period, 66% had abnormal Doppler velocimetry, and nearly 62% had abnormal cranial ultrasound in grayscale.

#### 3.2.3. Elastography abnormalities (shear-wave ultrasound-based)

The functional studies based on shear-wave elastography were performed in five regions of interest (ROIs) and “E” cut-off references were adopted according to previous recent literature (6–8), as there is no definitive normality parameter for elastography studies in the pediatric brain.

A significant relationship was found between maternal exposure to SARS-CoV-2 and elastography changes, mainly in the cerebral deep white matter and basal ganglia, in terms of stiffness alterations, with a decrease of the elastic modulus (*E*) in the SARS-CoV-2-exposed group when compared to controls. Table 4 shows these findings categorized by ROIs.

**Table 4:** Neuroimaging parameters of children distributed between the group exposed to SARS-CoV-2 infection during gestation (cases) and the non-exposed (control) group, according to the specific regions of interest (ROIs) for elastography analysis: deep white matter, frontal white matter, caudate/thalamus, corpus callosum, and frontal cortex.

Variable^a^	Groups		*p*-value*
	Cases (*n* = 201)	Control (*n* = 18)	
Elastography—DWM	3.98 ± 0.62	7.76 ± 0.77	<0.001
Elastography—FWM	3.31 ± 0.59	4.69 ± 0.85	<0.001
Elastography—caudate/thalamus	5.45 ± 0.64	6.46 ± 0.96	<0.001
Elastography—corpus callosum	4.53 ± 0.39	7.93 ± 0.88	<0.001
Elastography—frontal cortex	5.62 ± 0.57	6.59 ± 0.66	<0.001

DWM, deep white matter; FWM, frontal white matter.

^a^ Values expressed in kilopascal, as mean ± standard error.

^*^ *p*-value calculated by Mann-Whitney test.

The SARS-CoV-2 group had significantly lower “*E*” coefficients in specific brain areas, including the deep/periventricular white matter and the splenium of the corpus callosum. The basal ganglia (caudate nuclei and thalamus), superficial white matter, and cortical gray matter also showed stiffness variations associated with SARS-CoV-2 exposure, although to a lesser extent.

A significant dose-response relationship was found between exposure to SARS-CoV-2 during pregnancy and the presence of neuroimaging abnormalities, including grayscale, Doppler, and elastography modalities.

The neuroimaging parameter means were also compared between groups (SARS-CoV-2 exposure vs. non-exposure) using an analysis of covariance (ANCOVA) model. In this ANCOVA model, the neuroimaging parameter measures (hemodynamic indices and elastic modulus) were considered dependent variables, the group (SARS-CoV-2 exposure vs. non-exposure) was considered the independent variable, and the measures of GA (gestational age) and BW (birth weight) were considered covariates.

As shown in Table 5, the neuroimaging parameters of the patients present significant differences between the two groups, even after controlling for GA and BW. According to the data, the mean value of deep white matter elasticity in the group exposed to SARS-CoV-2 is 3.98 ± 0.04, while in the group without SARS-CoV-2, it is 7.77 ± 0.11. The difference between the two groups is −3.80 with a 95% confidence interval of [−4.03, −3.57] and a *p*-value of less than 0.001. This means that there is a statistically significant difference between the two groups for this parameter, indicating that patients exposed to SARS-CoV-2 during pregnancy have lower values for deep white matter elasticity compared to those not exposed to SARS-CoV-2. In contrast, the parameters for a single vessel (basilar artery) interestingly did not show a significant difference between the two groups, when adjusted for GA and BW. Considering the basilar artery RI, the difference between the two groups is −0.02 with a 95% confidence interval of [−0.04, 0.01] and a *p*-value of 0.2324.

**Table 5:** Neuroimaging parameters of infants distributed between the group exposed to SARS-CoV-2 infection during gestation (cases) and the unexposed group (control), controlled by GA (gestational age) and BW (birth weight), according to the specific regions of interest (ROIs) for elastography analysis: deep white matter, frontal white matter, caudate/thalamus, corpus callosum, and frontal cortex; *p*-value calculated by ANCOVA model.

Variable	Groups—mean value^a^ ± standard error		Comparison between groups	
	Cases (*n* = 201)	Control (*n* = 18)	Difference [CI 95%]	*p*-value*
MCA—RI	0.76 ± 0.01	0.79 ± 0.01	−0.03 [−0.07; −0.00]	0.0451
MCA—PI	1.59 ± 0.03	1.65 ± 0.08	−0.04 [−0.08; −0.00]	0.0434
Pericallosal artery—RI	0.70 ± 0.00	0.73 ± 0.01	−0.03 [−0.06; −0.00]	0.0277
Pericallosal artery—PI	1.26 ± 0.02	1.40 ± 0.05	−0.14 [−0.25; −0.03]	0.0123
Basilar artery—RI	0.71 ± 0.00	0.73 ± 0.02	−0.02 [−0.04; 0.01]	0.2324
Basilar artery—PI	1.31 ± 0.02	1.32 ± 0.07	−0.01 [−0.14; 0.14]	0.9585
Elastography—DWM	3.98 ± 0.04	7.77 ± 0.11	−3.80 [−4.03; −3.57]	<0.001
Elastography—FWM	3.31 ± 0.04	4.69 ± 0.11	−1.37 [−1.60; −1.14]	<0.001
Elastography—caudate nucleus/thalamus	5.46 ± 0.05	6.45 ± 0.12	−0.99 [−1.24; −0.74]	<0.001
Elastography—corpus callosum	4.56 ± 0.03	7.92 ± 0.08	−3.38 [−3.56; −3.20]	<0.001
Elastography—frontal cortex	5.61 ± 0.04	6.60 ± 0.10	−0.99 [−1.20; −0.78]	<0.001

Mean values adjusted by ANCOVA model. MCA, middle cerebral artery; RI, resistance index; PI, pulsatility index; DWM, deep white matter; FWM, frontal white matter.

^a^ Results are expressed in kilopascals, as mean ± standard error.

*p-values for comparison between groups were calculated using ANCOVA model, with GA and BW as covariates.

## 4. Discussion

### 4.1. General evidence

A systemic inflammatory response to the SARS-CoV-2 virus and consequent endothelial damage has been implicated in COVID-19 pathogenesis, with replicated evidence in many studies in both biochemical and clinical settings (9–11). Although there is extensive epidemiologic evidence of systemic COVID-19 effects (12, 13), the neurologic consequences of SARS-CoV-2 exposure in the pediatric group are still uncertain, and current evidence is mostly based on case reports (14, 15). It is not clear whether and to what extent the blood-brain barrier functions as a protective factor in blocking inflammatory cytokines (16–19).

Our study provides evidence that SARS-CoV-2 infection during pregnancy may be associated with both structural and functional brain damage in infants. The most recurrent findings were characterized in the cerebral deep white matter, although all other ROIs demonstrated some degree of change. These changes were manifested by increased regional echogenicity on B-mode studies, a reduction in the corresponding resistance/pulsatility of intracranial arterial flow, and a decrease in the cerebral elastic modulus. The reduced stiffness in the cerebral tissue, especially in the deep white matter, may represent a decreased amount of tissular myelin in the central nervous system, a crucial element for adequate neurodevelopment in children. Few neuroimaging studies have been conducted in this area with pediatric subjects, so our results provide unprecedented evidence based on structural and functional abnormalities.

### 4.2. Ultrasonographic findings (gray scale)

Structural neuroimaging scans in our study have repeatedly demonstrated white matter involvement in abnormal cases in SARS-CoV-2-exposed subjects. To date, there are published case series (20, 21) reporting a similar pattern of involvement in COVID-19, but no longitudinally designed studies with SARS-CoV-2-exposed and unexposed control groups correlating neuroimaging findings and clinical follow-up.

Because there is exceptional collateral circulation in the brain vasculature in the neonatal period and early childhood, the pattern of parenchymal involvement in these subjects tends to be less severe in the cortical gray matter (unlike in adults). In response to vascular and/or hypoxic encephalic injury, the deep white matter is one of the first areas of the brain affected during this early period of life (22–24).

This evidence was replicated in our results, as both deep white matter and basal ganglia areas presented as regions of higher echogenicity in abnormal B-mode scans when compared to controls (the unexposed group). In our sample, 18 individuals whose mothers were infected by SARS-CoV-2 during pregnancy manifested some degree of white matter disease, of which 16 (88.8%) had exclusive white matter involvement and two (11.2%) subjects had concomitant involvement of deep white matter and cerebral basal ganglia (thalami and caudate nuclei). Another significant finding of the morphometric US studies was the persistence of increased echogenicity in the affected areas at the routine follow-up ultrasound study, performed 4 weeks after the initial scan, in all the abnormal cases. At re-evaluation, it was possible to characterize an increase in the extent of the affected areas in three individuals (16.6%) who evolved from initial exclusive deep white matter lesions to additional abnormalities in the basal ganglia, in total, the caudate nuclei and thalami.

Although the correlation of basal ganglia changes with the clinical COVID-19 syndrome is still unclear, it is thought to play a role in the long-lasting damage that some infants  have shown, manifesting as late-onset post-COVID-19 symptoms, with delayed neurological development and failure to achieve neuropsychomotor milestones at specific ages (25, 26).

### 4.3. Hemodynamics findings (Doppler evaluation)

Our data regarding intracranial blood flow analysis in both groups suggest a relevant trend of decrease in RI (resistance) and PI (pulsatility) indices in the SARS-CoV-2-exposed group when maternal infections occur in the last 14 days of gestation and critical cases. This fact is thought to be a consequence of systemic adaptation to the persistent inflammatory condition that may be present even after the first 14 days of acute viral symptoms (27, 28). Cases of early maternal infection with SARS-CoV-2 during pregnancy, especially in the first and second trimesters, would allow sufficient time for arterial flow autoregulation to settle and the systemic inflammatory response to subside.

Such hemodynamic adaptation findings have been widely reported in the literature for other conditions predisposing to brain injury, such as hypoxic-ischemic injury, metabolic damage, and systemic inflammatory conditions (SIRS—systemic inflammatory response syndrome) (29, 30), generally indicating situations in which the brain has increased metabolic demands and a significant increase in intracranial blood flow is required. In fetal life, an analogous situation is classically demonstrated in cases of fetal intrauterine growth-restriction (IUGR), when the fetal arterial flow is redirected to the intracranial circulation to the detriment of visceral and peripheral flow (31–33).

Unlike other viral infections with the well-known transplacental transmission, such as human cytomegalovirus (CMV), rubella virus, parvovirus B19, and Zika virus (ZIKV), the worst pregnancy outcomes in SARS-CoV-2 infection were observed in late-stage pregnancies. This finding is consistent with the current literature, as current evidence does not demonstrate that SARS-CoV-2 represents efficient transplacental virus transmission or direct fetal neuronal damage (34, 35).

### 4.4. Elastography findings (shear-wave elastography assessment)

To our knowledge, no previous study has assessed elastography parameters of the brain parenchyma in infants exposed to SARS-CoV-2 during pregnancy. The few publications in the pediatric literature include small case series of healthy individuals aimed at suggesting standard elastography values for normal brain parenchyma in neonates (36, 37). Other similar studies have been conducted in mice with anatomopathological correlations (38, 39). Experiments in mice achieved a significant level of agreement with human brain values, presumably related to the very similar elasticity coefficients/energy densities (*p*) of mouse and human brains.

When the elastography data of our study groups were analyzed, significant differences were found between SARS-CoV-2-exposed newborns and the unexposed group in terms of the elastic modulus of the brain parenchyma. All regions of interest (ROIs) showed a reduction in the elasticity coefficient/Young's modulus (*E*) in the SARS-CoV-2-exposed group.

The elastography pattern differences between both groups were more pronounced in the DWM deep white matter zone (ROI number 1) when compared to other regions of analysis such as subcortical white matter and the frontal cortex. A plausible hypothesis is related to differences in the tissular composition of these regions, with a predominance of myelin in the deep white matter (40). Considering also the age of the subjects (6 months of adjusted chronological age), our ROI at the DWM was expected to be myelinated at this stage, different from the subcortex or frontal cortical zones (41–43). These elements suggest that brain findings related to SARS-CoV-2 exposure during pregnancy may be due, to some extent, to changes in the amount of myelin in the cerebral tissue, knowing that those with less myelin present a decrease in their elasticity coefficients, corresponding to a reduction in stiffness. Another possible mechanism could be mild intra-myelinic edema, in which the inflammation causes an increased water content in the cerebral tissue, thus leading to a decrease in tissue stiffness.

Our findings are consistent with recent studies investigating the impact of SARS-CoV-2 infection during pregnancy on pediatric neurodevelopment. Regarding neuroimaging, a study published in October 2021 aimed to assess the association between maternal SARS-CoV-2 infection during pregnancy and offspring brain development using MRI scans (44). The study followed 55 infants born to mothers with SARS-CoV-2 infection during pregnancy. The researchers found that infants born to mothers with SARS-CoV-2 infection during pregnancy had reduced cortical thickness in the left superior temporal gyrus, which is an important brain region for language and social communication. Abnormal cortical thickness in this region has been associated with neurodevelopmental conditions such as autism spectrum disorders. The study suggests that maternal SARS-CoV-2 infection during pregnancy may affect offspring brain development, particularly in brain regions important for language and social communication.

Protocols with a more clinical focus included a study published in January 2022 (45) that followed 205 children born to mothers with SARS-CoV-2 infection during pregnancy and found that children born to mothers with SARS-CoV-2 infection during pregnancy had an increased risk of developmental delay at 12 months of age compared to children born to mothers without SARS-CoV-2 infection. Another study published in August 2021 (46) found that children born to mothers with severe or critical COVID-19 during pregnancy had a higher risk of cognitive, motor, and language developmental delays at 6 months of age compared to children born to mothers without COVID-19. The study followed 150 infants born to mothers with COVID-19 and 150 infants born to mothers without COVID-19.

It is worth noting that these studies have limitations, and more research is needed to fully understand the potential effects of SARS-CoV-2 infection during pregnancy on pediatric neurodevelopment. However, the findings suggest that healthcare providers should closely monitor children born to mothers with SARS-CoV-2 infection during pregnancy for any signs of developmental delays or neurodevelopmental disorders.

### 4.5. Limitations

Causal associations between SARS-CoV-2 infection and adverse perinatal outcomes have been suggested in clinical studies but have not been definitely established, as there are many potential confounding factors involved. Among these, we should emphasize that mothers infected by SARS-CoV-2 during pregnancy are often prone to gestational complications, including adverse birth conditions, preterm labor, and maternal and neonatal hypoxia—factors that may themselves lead to CNS damage. Controlling all of these factors can be challenging. Our study attempted to control for some of these possible biases with covariance analysis techniques. However, many characteristics related to clinical maternal status, such as gestational hypertension, diabetes, previous lung disease, and obesity, persisted in our sample for both groups and may affect neurodevelopmental outcomes in infants. It is also relevant to consider that most of our sample was composed of outpatients, so the severity of maternal infection was predominantly mild to moderate, and there was a quantitative disproportion between case and control groups, given the context of multiple lockdowns and subsequent SARS-CoV-2 vaccination—the latter being one of the exclusion criteria for the control group. We acknowledge the substantial difference in the sample size between the cases and controls, and the possibility of introducing bias as a result. Because infant outcomes of maternal SARS-CoV-2 exposure during pregnancy are poorly defined to date, an accurate prospective sample size estimation for cases and controls was not feasible. However, a *post hoc* analysis was performed to estimate the number of controls needed to maintain a probability of error (alpha) of 0.05 with a power of 0.8 using the relative frequency of abnormal imaging findings in the cases. We used a likelihood ratio test to estimate the sample size needed for controls and found that *N* = 9. Thus, we believe that the control group in our study is sufficient for our research questions. Furthermore, the prospective recruitment of our controls involved randomly selecting individuals from a large representative population in our universal public health system.

## 5. Conclusion

SARS-CoV-2 infection during pregnancy is associated with encephalic changes in a relevant proportion of cases, predominantly affecting the cerebral deep white matter (DWM). The characteristic SARS-CoV-2-related pediatric leukopathy is manifested in neuroimaging with increased echogenicity and decreased elasticity coefficients in the DWM, i.e., reduced stiffness. These findings open up a spectrum of research possibilities regarding their effects on fetal, neonatal, and childhood health. The description of the consequences of infection in long-term follow-up may provide a better understanding of the disease and its impact on the central nervous system.

Future research using correlated axial methods, such as magnetic resonance imaging and tractography, may contribute to predicting brain areas more vulnerable to SARS-CoV-2-related encephalopathy and delineating regions with a propensity for decreased myelination. By understanding the neuroimaging correlates of SARS-CoV-2 infection in the perinatal period, this study could provide a more complete picture of the presentation pattern in the brain of SARS-CoV-2-exposed individuals during early childhood. The characterization of pediatric brain areas with a higher risk of neurological damage following maternal SARS-CoV-2 infection will allow the evaluation of clinical correlates and the early prevention of neurodevelopmental sequelae.

## Data Availability

The original contributions presented in the study are included in the article/**Supplementary Material**, further inquiries can be directed to the corresponding author.
