# Supplementary material for: Neuroimaging assessment of pediatric cerebral changes associated with SARS-CoV-2 infection during pregnancy
Source: Front Pediatr. 2023 May 24;11:1194114. doi: 10.3389/fped.2023.1194114 (PMC10244818; doi:10.3389/fped.2023.1194114)
Supplement: Supplementary file 1 [file Datasheet1.docx]

**APPENDIX A -**

**Acquisition of color Doppler and spectral images, measurement of hemodynamic indices in the main intracranial arteries**

**
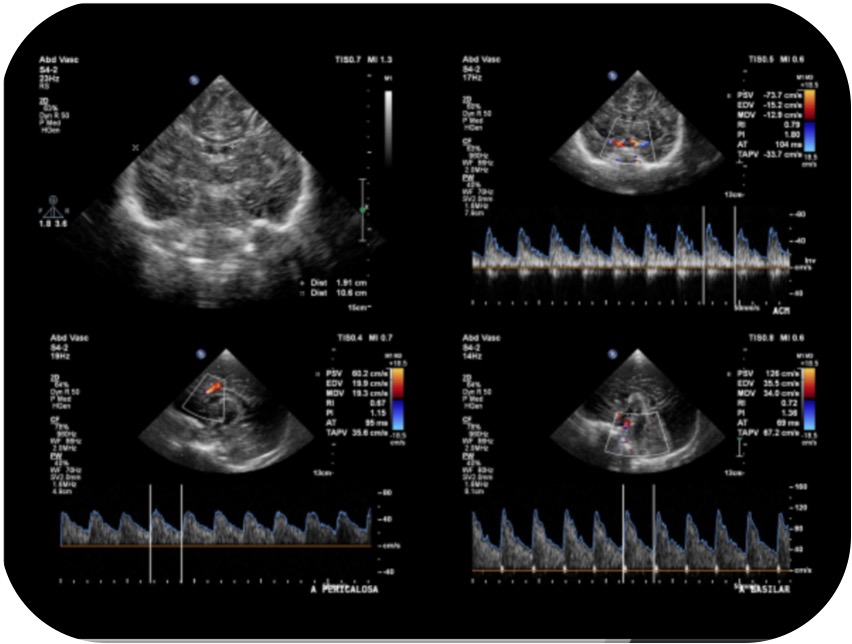
**

Figure A1. Transcranial ultrasonography images in B-mode (grayscale) coronal view for evaluation of the deep white matter over the anterior fontanelle; and Doppler flowmetry of the major intracranial arteries.


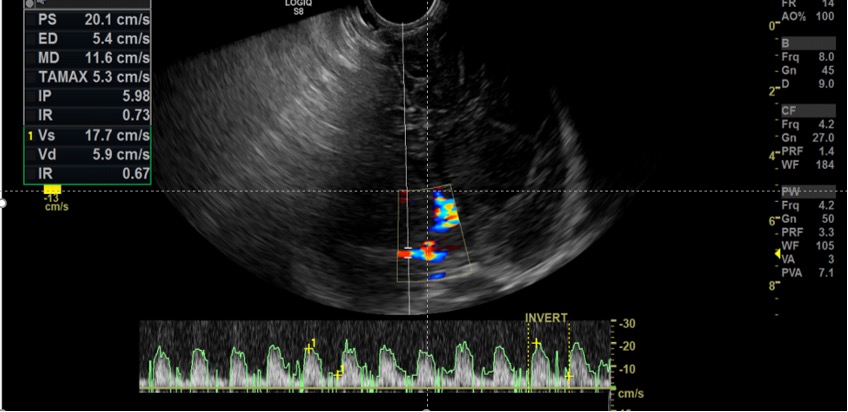


Figure A2. Transcranial ultrasonography images in B-mode (grayscale) coronal view over the anterior fontanelle, with color and spectral Doppler for evaluation of hemodynamic indices in the middle cerebral artery.


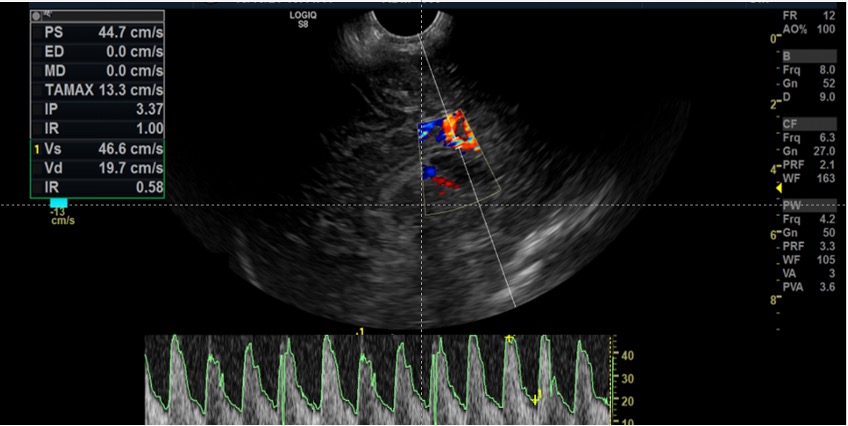


Figure A3. Transcranial ultrasonography images in B-mode (grayscale) sagittal view over the anterior fontanelle, with color and spectral Doppler for evaluation of hemodynamic indices in the pericallosal artery.


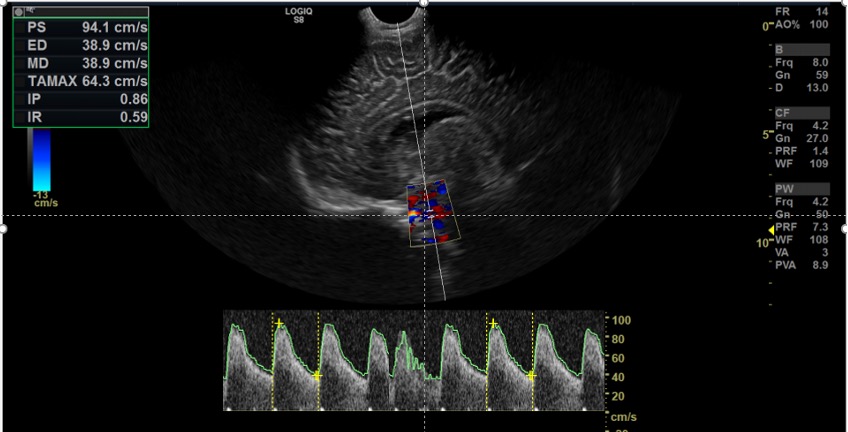


Figure A4*.* Transcranial ultrasonography images in B-mode (grayscale) coronal view over the anterior fontanelle, with color and spectral Doppler for evaluation of hemodynamic indices in the basilar artery. Velocities are expressed in cm/s, dimensionless IR, and IP indices.
