# Supplementary material for: Neuroimaging assessment of pediatric cerebral changes associated with SARS-CoV-2 infection during pregnancy
Source: Front Pediatr. 2023 May 24;11:1194114. doi: 10.3389/fped.2023.1194114 (PMC10244818; doi:10.3389/fped.2023.1194114)
Supplement: Supplementary file 2 [file Datasheet2.docx]

**APPENDIX B –**

**Acquisition of gray scale images for real time fusion with elastography map (Shear Wave Mode) and demonstration of module “E” measurements acquired in regions of interest (ROIs) in the brain.**

**
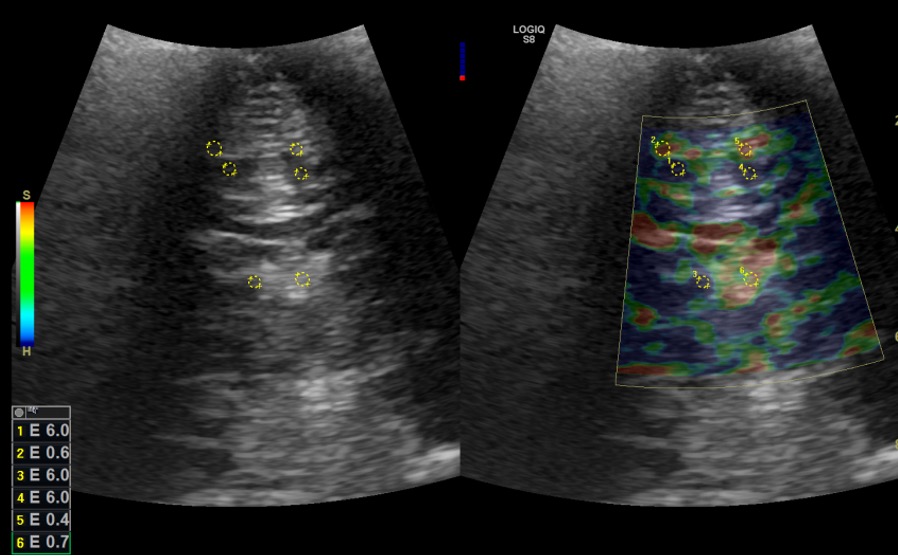
**

Figure B1. Shear-wave US elastography with color map in the region of the basal ganglia and deep white matter, demonstrating the acquisition of measurements in six regions of interest (circular ROIs), in the coronal plane. Elastic modulus measurements in the regions of interest expressed in kilopascals (kPa).


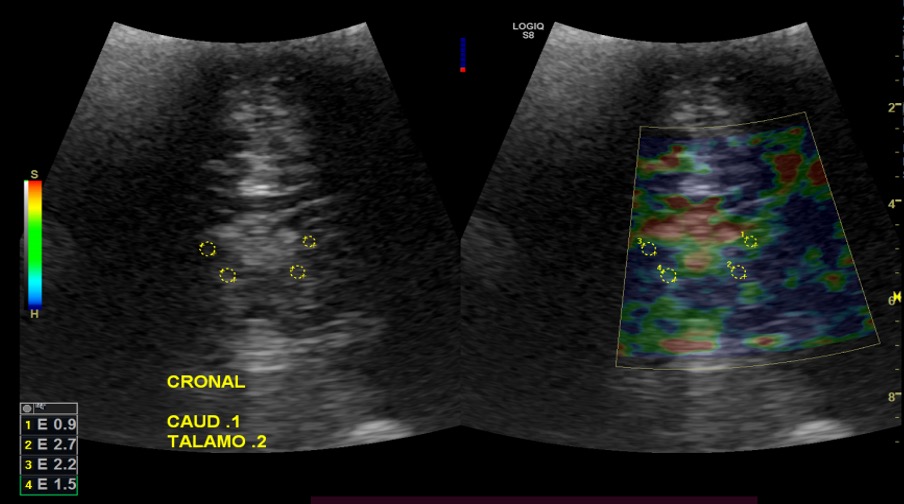


Figure B2. Shear-wave US elastography with color map in the region of the thalamus and caudate nuclei, demonstrating the acquisition of measurements in four regions of interest (circular ROIs), in the coronal plane. Elastic modulus measurements in the regions of interest expressed in kilopascals (kPa).

**
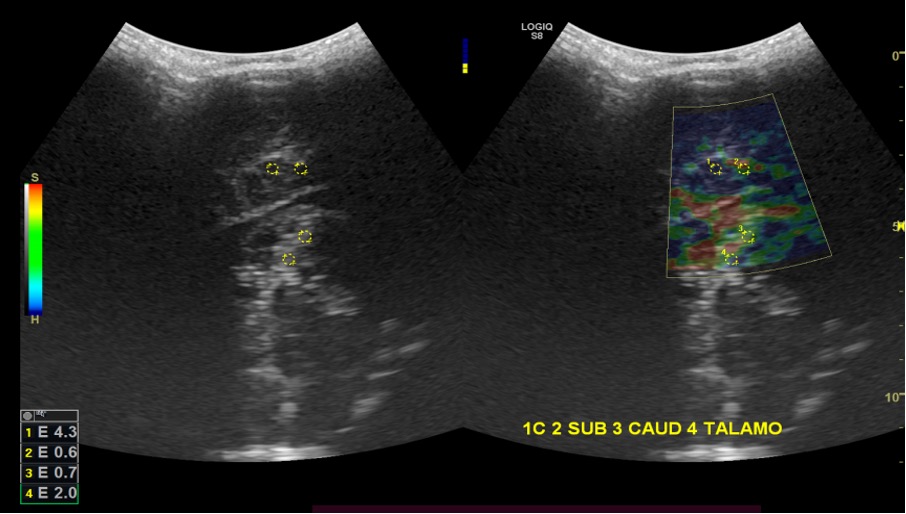
**

Figure B3. Shear-wave US elastography with color map in the region of the cortex, subcortical white matter, caudate nucleus, and thalamus, demonstrating the acquisition of measurements in four regions of interest (circular ROIs), in the sagittal plane. Elastic modulus measurements in the regions of interest expressed in kilopascals (kPa).

**
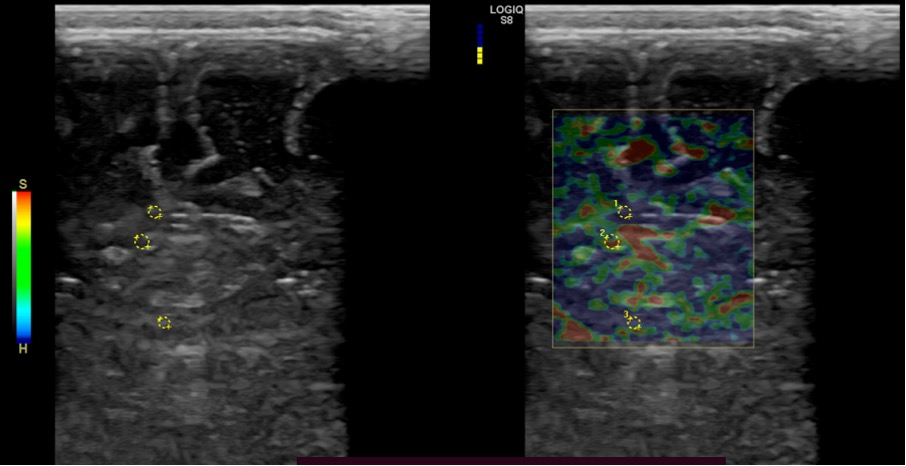
**

Figure B4. Shear-wave US elastography with color map in the cortico-subcortical interface region, including frontal cortical gray matter, juxtacortical white matter, and deep white matter, demonstrating the acquisition of measurements in three regions of interest (circular ROIs), in the coronal plane. Elastic modulus measurements in the regions of interest expressed in kilopascals (kPa).


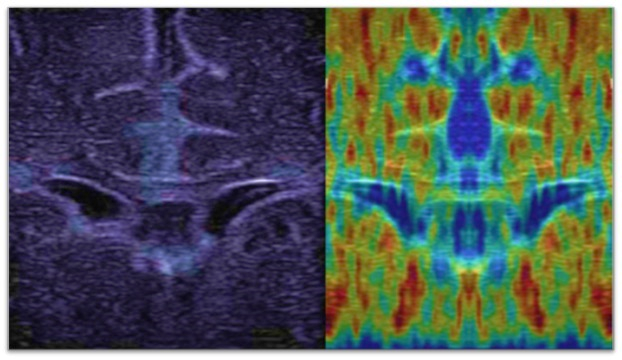


Figure B5. Shear-wave mode US elastography with color map in the cortical-subcortical interface region, including frontal cortical gray matter, juxtacortical white matter, deep white matter, and basal ganglia, showing the variation of the color map according to the elastic modulus of (E) in a patient whose mother was exposed to SARS-Cov-2 infection during pregnancy, with reduced elasticity affecting the basal ganglia bilaterally (areas shown in blue) and some zones of the deep white matter. Other zones in blue correspond to CSF circulation spaces, with mostly low “E” values.
