# Supplementary material for: Neuroimaging assessment of pediatric cerebral changes associated with SARS-CoV-2 infection during pregnancy
Source: Front Pediatr. 2023 May 24;11:1194114. doi: 10.3389/fped.2023.1194114 (PMC10244818; doi:10.3389/fped.2023.1194114)
Supplement: Supplementary file 3 [file Datasheet3.docx]

**APPENDIX C –**

**Supplementary Table 1C. Maternal and pediatric clinical conditions/comorbidities distributed between the group exposed to SARS-CoV-2 infection during pregnancy (cases) and the unexposed group (control), according to pre- and post-natal assessments at outpatient clinics.**

|  | Groups | |  |
| --- | --- | --- | --- |
| Maternal diseases^*^ | **Cases (n= 201)** | **Control (n = 18)** | **p-value^#^** |
| Systemic arterial hypertension | 7.4% (15) | 5.5% (1) | 0.1187 |
| Pregestational diabetes | 6.9% (14) | 5.5% (1) | 0.1174 |
| Pulmonary disease | 7.4% (15) | 0 | 0.1335 |
| Cardiac disease | 2.9% (6) | 0 | 0.1208 |
| Obesity | 10.4% (21) | 5.5% (1) | 0.1276 |
|  | **Groups** | |  |
| Infant diseases/comorbidities^&^ | **Cases (n= 201)** | **Control (n = 18)** | **p-value^#^** |
| Anemia | 18.9% (38) | 11.1% (2) | 0.1420 |
| Malnutrition | 4.9% (10) | 5.5% (1) | 0.1123 |
| Obesity | 2.4% (5) | 0 | 0.1196 |
| Bronchospasm | 7.9% (16) | 5.5% (1) | 0.1201 |
| Miscellaneous | 22.3% (45) | 16.6% (3) | 0.1371 |

* values expressed as percentage and frequency, as % (n).

# p-value calculated by Fisher’s test. Level of significance at 0.05 (p-values>0.05 - no significant difference between groups).

^&^Conditions diagnosed during the first 6 months of clinical follow-up. Anemia was defined as hemoglobin <11 g/dL, undernutrition was defined as weight-for-age z-score <-2 SD, obesity was defined as weight-for-length z-score >2 SD, and bronchospasm was defined as a physician diagnosis of asthma or wheezing. The miscellaneous category included: rhinitis, dermatitis, cow’s milk protein allergy (CMPA), and gastroesophageal reflux disease (GERD).
